# Evaluating oral health-related quality of life measure for children and preadolescents with temporomandibular disorder

**DOI:** 10.1186/1477-7525-9-32

**Published:** 2011-05-12

**Authors:** Taís S Barbosa, Marina S Leme, Paula M Castelo, Maria Beatriz D Gavião

**Affiliations:** 1 Department of Pediatric Dentistry, Piracicaba Dental School, State University of Campinas, Piracicaba/SP, Brazil; 2 Department of Biological Sciences, Federal University of São Paulo, Diadema/SP, Brazil

## Abstract

**Background:**

Oral health-related quality of life (OHRQoL) in children and adolescents with signs and symptoms of temporomandibular disorder (TMD) has not yet been measured. This study aimed to evaluate the validity and reliability of OHRQoL measure for use in children and preadolescents with signs and symptoms of TMD.

**Methods:**

Five hundred and forty-seven students aged 8-14 years were recruited from public schools in Piracicaba, Brazil. Self-perceptions of QoL were measured using the Brazilian Portuguese versions of Child Perceptions Questionnaires (CPQ)_8-10 _(n = 247) and CPQ_11-14 _(n = 300). A single examiner, trained and calibrated for diagnosis according to the Axis I of the Research Diagnostic Criteria for TMD (RDC/TMD), examined the participants. A self-report questionnaire assessed subjective symptoms of TMD. Intraexaminer reliability was assessed for the RDC/TMD clinical examinations using Cohen's Kappa (*κ*) and intraclass correlation coefficient (ICC). Criterion validity was calculated using the Spearman's correlation, construct validity using the Spearman's correlation and the Mann-Whitney test, and the magnitude of the difference between groups using effect size (ES). Reliability was determined using Cronbach's alpha, alpha if the item was deleted and corrected item-total correlation.

**Results:**

Intraexaminer reliability values ranged from regular (*κ *= 0.30) to excellent (*κ *= 0.96) for the categorical variables and from moderate (ICC = 0.49) to substantial (ICC = 0.74) for the continuous variables. Criterion validity was supported by significant associations between both CPQ scores and pain-related questions for the TMD groups. Mean CPQ_8-10 _scores were slightly higher for TMD children than control children (ES = 0.43). Preadolescents with TMD had moderately higher scores than the control ones (ES = 0.62; p < 0.0001). Significant correlation between the CPQ scores and global oral health, as well as overall well-being ratings (p < 0.001) occurred, supporting the construct validity. The Cronbach's alphas were 0.93 for CPQ_8-10 _and 0.94 for CPQ_11-14_. For the overall CPQ_8-10 _and CPQ_11-14 _scales, the corrected item-total correlation coefficients ranged from 0.39-0.76 and from 0.28-0.73, respectively. The alpha coefficients did not increase when any of the items were deleted in either CPQ samples.

**Conclusions:**

The questionnaires are valid and reliable for use in children and preadolescents with signs and symptoms of temporomandibular disorder.

## Introduction

Over the years, different theories of etiology and different emphases on the causative factors for the various signs and symptoms of temporomandibular disorder (TMD) have been proposed in the literature [1]. The current perspective regarding TMD is now multidimensional, with an appreciation that a combination of physical, psychological and social factors may contribute to the overall presentation of this disorder. Hence, today there is a preference for a biopsychosocial integrated approach [2]. Accordingly, TMD patients are a target population for quality of life (QoL) assessments because of the considerable psychosocial impact of orofacial pain [3]. TMD have generally been presumed to be conditions affecting only adults; however, epidemiological studies have reported signs and symptoms in children and adolescents to be as frequent as in adults [4] and the prevalence varies widely in the literature from 16% to 90%, due to the methodologies focusing largely on samples of patients seeking treatment or because they were conducted on convenience non-representative samples of the population. Brazilian studies have shown that in primary dentition 34% of the 99 children presented at least one sign and/or one symptom of TMD [5]. In the age of 12 years, 2.19% of the boys and 8.18% of the girls met the Research Diagnostic Criteria for TMD (RDC/TMD) when examined [6]. From 15 to 20 years-old 35.4% presented at least one symptom of TMD [7]. Signs and symptoms in childhood and adolescence have been indicating mild disorders, but these findings do not detract from the importance of early diagnosis to provide proper growth and development of the stomatognathic system [8]. Additionally the known fluctuation in signs and symptoms of musculoskeletal disorders in a time-dependent context might have been better addressed by carrying out repeated clinical recordings [4]. In addition, Dahlström and Carlsson [9], in a recent systematic review, observed a substantial negative impact on oral health-related quality of life (OHRQoL) in patients diagnosed with TMDs, being greater than other orofacial diseases/illnesses or conditions.

In this way, measuring health-related quality of life (HRQoL) in TMD patients with generic or condition-specific HRQoL instruments can complement efficacy measures, offering a complete picture of the impact of disease and treatment on overall well-being, as observed in adolescents with type 1 diabetes [10]. Jedel *et al. *[11] compared the HRQoL between children with TMD pain and a control group, using the Child health questionnaire-child form 87 (CHQ-CF87), a generic multidimensional instrument designed to assess physical and psychosocial impacts on children and adolescents aged 10-18 years. Although the results supported the use of generic instrument to measure health and to evaluate the efficacy of treatment in pediatric patients with TMD pain [11], other authors recommend the use of condition-specific instruments, which are more sensitive for detecting slight changes in specific conditions [12] and might allow a more detailed evaluation of the disability caused by TMD [13]. Accordingly, studies were conducted to evaluate the impact of TMD and associated pain on QoL in adult [3,12,14,15] and elderly [16] populations, using a condition-specific instruments, i.e., an OHRQoL measure (e.g., Oral Health Impact Profile and Geriatric Oral Health Assessment Index). The concepts in OHRQoL provide an opportunity to summarize a variety of possible psychosocial impacts in relation to specific oral diseases [14].

Measures have been developed specifically for assessing OHRQoL of children and adolescents [17-21]. The Child Perceptions Questionnaire (CPQ) is a measure applicable to children with a wide variety of oral and orofacial conditions, based on contemporary concepts of pediatric health and which can accommodate developmental differences among children across age ranges [17,18]. It consists of two age specific instruments for children aged 8-10 years (CPQ_8-10_) [18] and 11-14 years (CPQ_11-14_) [17]. A preliminary study has confirmed the validity and reliability of these measures for use in Brazilian children and adolescents [22]. Although these questionnaires are standardized and widely used for other oral conditions, they have not yet been tested in TMD samples.

Assessing the impact of TMD on children's QoL is important in many fronts. It provides an insight into the potential consequences of TMD to the day-to-day lives of children and thereby facilitates understanding of its importance in the provision of oral health care [23]. Moreover, identifying factors associated with the impact of TMD on children's QoL can influence management of such cases and inform best practice guidelines [24]. In this way, the present study aimed to test the validity and reliability of CPQ used in a population of Brazilian public school students aged 8-14 years to determine whether these measures are sensitive to clinical signs and subjective symptoms of TMD. An additional aim was to verify whether the presence and severity of signs and symptoms of TMD are sufficient to influence OHRQoL of this age-specific population.

## Material and methods

This study was approved by the Research Ethics Committee of the Dental School of Piracicaba, State University of Campinas (protocol n°021/2006).

A cross-sectional study with students of public schools of Piracicaba, Brazil, was developed. Piracicaba city has 368.843 scholars, with 50.187 enrolled in the elementary school system http://www.ibge.gov.br. The sample size was calculated by Epi info version 6.0.1 software. A standard error of 2%, a 95% confidence interval level and a 5.73% prevalence of TMD [25] were used for the calculation. The minimum sample size to satisfy the requirements was estimated at 513 subjects. A total of 547 students (235 boys and 312 girls), with no systemic diseases or communication and/or neuromuscular problems, participated in the study. The subjects ranged from 8 to 14 years of age, and were from nine public schools, which were randomly selected. All students obtained parental consent.

The exclusion criteria were conditions/children with facial traumatism, neurological or psychiatric disorders, use of dental prostheses, current use of medications (e.g., antidepressive, muscle relaxant, narcotic or non-steroidal anti-inflammatory), previous or present orthodontic treatment and other orofacial pain conditions, which could interfere with TMD diagnoses.

### Data collection

#### Oral health-related quality of life evaluation

Data were collected using the Portuguese versions of the CPQ for individuals aged 8-10 years (CPQ_8-10_) and 11-14 years (CPQ_11-14_) [22]. These formed the components of the Child Oral Health Quality of Life Questionnaire that had been designed to assess the impact of oral conditions on the QoL of children and adolescents [17,18]. They were both self-completed. Items of the CPQ used Likert-type scales with response options of "Never" = 0; "Once or twice" = 1; "Sometimes" = 2; "Often" = 3; and "Very often" = 4. For the CPQ_11-14_, the recall period was three months, while for that of the CPQ_8-10_, it was four weeks. Items were grouped into four domains: oral symptoms, functional limitations, emotional well-being and social well-being.

Children and adolescents were also asked to give overall or global assessments of their oral health and the extent to which the oral or oro-facial condition affected their overall well-being. These questions preceded the multi-item scales in the questionnaires. A four-point response format, ranging from "Very good" = 0 to "Poor" and from "Not at all" = 0 to "A lot" = 3, was offered for these ratings in CPQ_8-10_. In CPQ_11-14_, these global ratings had a five-point response format ranging from "Excellent" = 0 to "Poor" = 5 for oral health and from "Not at all" = 0 to "Very much" = 5 for well-being.

#### Evaluation of signs and symptoms of TMD

##### Intraexaminer reliability

Prior to the clinical examinations, the dental examiner (TSB) participated in the calibration process, which was divided into theoretical discussions on codes and criteria for the study, as well as practical activities. Intra-examiner reliability was investigated by conducting replicated examinations on 20 individuals one week later to minimize recall bias as a result of the first test.

##### RDC/TMD

The RDC/TMD is a classification system composed by a dual-axis approach: Axis I (physical findings) and Axis II (pain-related disability and psychosocial status).

##### Subjective symptom interview

A self-report questionnaire was used to assess subjective symptoms according to Riolo *et al. *[26], regarding pain in the jaws when functioning (e.g., chewing), unusually frequent headaches (i.e., more than once a week and of unknown etiology), stiffness/tiredness in the jaws, difficulty opening one's mouth, grinding of the teeth and sounds from the TMJ. Each question could be answered with a "yes" or a "no."

Moreover, three specific questions (yes/no) of the RDC/TMD Axis II were considered for further TMD diagnosis [27,28]: (1) *Have you had pain in the face, jaw, temple, in front of the ear or in the ear in the past month?*; (2) *Have you ever had your jaw lock or catch so that it won't open all the way?*; (3) *Was this limitation in jaw opening severe enough to interfere with your ability to eat? *The other questions of Axis II were not included due to difficulty to understand or inappropriate for children.

##### Clinical signs evaluation

The clinical signs of TMD were assessed using the RDC/TMD criteria (Axis I) described as follows [28,29]:

*Pain Site*. To determine whether the present pain was ipsilateral to the pain provoked by the clinical examination of the masticatory muscles and during jaw function.

*Mandibular Range of Motion (mm) and Associated Pain*. Jaw-opening patterns. Corrected and uncorrected deviations in jaw excursions during vertical jaw opening. Vertical range of motion of the mandible. Extent of unassisted opening without pain, maximum unassisted opening and maximum assisted opening. Mandibular excursive movements. Extent of lateral and protrusive jaw excursions.

*Temporomandibular Joint Sounds*. Palpation of the TMJ for clicking, grating, and crepitus sounds during vertical, lateral and protrusive jaw excursions.

*Muscle and Joint Palpation for Tenderness*. Bilateral palpation of extraoral and intraoral masticatory and related muscles (n = 20 sites) and bilateral palpation of the TMJ (n = 4 joint sites).

The clinical evaluation selected individuals with at least one sign and one symptom of TMD [30], who were referred to as the TMD group in this present study. Subjects meeting the criteria for myofascial pain with or without limited opening (Axis I, Group 1a or 1b disorders) and/or for disc displacement with reduction, without reduction with limited opening or without reduction without limited opening (Axis I, Group 2a, 2b or 2c) or for arthralgia or arthritis (Axis I, Group 3a or 3b) were considered to have an RDC/TMD diagnosis (RDC/TMD diagnosis group) [28]. The control group consisted of individuals with no current signs or symptoms of TMD (supercontrols) or those without signs or symptoms of TMD (control group) [14,28]. This recruitment strategy was based on the principle that subjects belonging to different groups will almost certainly respond differently to the questionnaire [31]. If the questionnaire is valid, it must be sensitive to such differences.

### Data analysis

Statistical analyses were performed using SPSS 9.0 (SPSS, Chicago, IL, USA) with a 5% significance level and normality was assessed using the Kolmogorov-Smirnov test. Since score distributions were asymmetrical, non-parametrical tests were used in the performed analyses.

Overall scores for each participant were calculated by summing the item codes, whereas the subscale scores were obtained by summing the codes for questions within the four health domains. Descriptive statistics were followed by bivariate analyses, which used (where appropriate) Chi-squared and Fisher's exact tests for a comparison of proportions and Mann-Whitney test for a comparison of the means of the continuous variables.

#### Intraexaminer reliability

Intraexaminer reliability calculations were performed on 20 individuals who participated in the Axis I assessment and the Axis II diagnosis interview. Only three questions (3, 14a, 14b) from the latter were used as required determinants for the Axis I diagnoses.

The two most commonly accepted methods for assessing the intraexaminer reliability were used [32]. When the clinical examination variable could be measured on a continuous scale, reliability was assessed by computing the intraclass correlation coefficient (ICC), using the one-way analysis of variance random effect parallel model [33]. The strength of the intra-examiner agreement was based on the following standards for ICC: < 0.2, poor; 0.21-0.40, fair; 0.41-0.60, moderate; 0.61-0.80, substantial and 0.81-1.0, excellent to perfect [34]. The Kappa statistic (Cohen's Kappa, *κ*) was computed to assess the reliability when variables were measured with a categorical rating scale (e.g., yes/no). Kappa values above 0.8 were considered excellent, from 0.61 to 0.8 good, 0.41 to 0.6 acceptable, 0.21 to 0.40 regular and below 0.20 fair [35].

#### Validity

The validity of a questionnaire represents the degree to which it measures what it is meant to measure. Criterion validity was calculated by comparing the correlations between CPQ scores and pain scores (obtained from Question 3 of the RDC/TMD Axis II), using the Spearman's correlation coefficient. As pain was considered a variable only in the TMD patients, the relevant correlation coefficients were calculated only for the TMD groups.

Discriminant construct validity was evaluated by comparing the mean scale scores between TMD and control groups using the Mann-Whitney test. The magnitude of the difference between groups was assessed using the effect size (ES). This was derived from the mean difference in scores between the groups divided by the pooled SD of scores: a value of 0.2 was taken to be small, 0.5 to be moderate and 0.8 to be large [36]. Discriminant construct validity was also assessed by verifying the difference between RDC/TMD diagnosis (individuals in Group I, II or III diagnosis) and "supercontrol" groups (individuals with no current sign and symptom of TMD). Correlational construct validity was assessed by comparing the mean scores and global ratings of oral health and overall well-being using Spearman's correlation coefficient.

#### Internal reliability

Reliability can be defined as a measure of the internal consistency or homogeneity of the items. Two measures were used for the analysis of internal reliability; the corrected item total correlation and the Cronbach's alpha coefficient [37]. Values above 0.2 for the former and 0.7 for the latter can be acceptable [38]. Alphas were also calculated with each item deleted.

## Results

### Descriptive statistics

A sample distribution of the evaluated characteristics (e.g., age, gender, TMD groups and CPQ scores) is shown in Additional file 1. Female children and preadolescents were more prevalent in TMD groups. Muscle tenderness and headaches were the most frequent signs and symptoms of TMD found in children and preadolescents, being observed more significantly in girls than in boys (Chi-squared test).

### Intraexaminer reliability

Among the 20 subjects for the reliability study, there were 14 girls and 6 boys with an average age of 10.30 ± 1.78 years. Fourteen of the subjects complained of symptoms suggestive of TMD, while six were asymptomatic. In almost all subjects (n = 19), at least one sign of TMD was observed. The frequency of individuals with RDC/TMD diagnosis was 10% for muscle tenderness and 5% for disc displacements, respectively.

Table 1 shows the intraexaminer reliability for the clinical examinations and diagnostic questions of RDC/TMD. The ICC and Kappa values for the former ranged from 0.49 to 0.74, indicating a moderate to substantial agreement and from 0.30 to 0.96, indicating a regular to excellent agreement, respectively. High levels of reliability were found for all three questions of the Axis II, with kappa values ranging from 0.70 to 0.81.

**Table 1:** Intraexaminer reliability of diagnostic questions and clinical examinations of the RDC/TMD criteria (n = 20)

	Reliability	
	
RDC/TMD criteria	Statistical tests	Interpretation
*Sign of TMD - Axis I*		
*Muscle tenderness*		
Extraoral myofascial sites (4-category variable)^†^	0.74	Substantial agreement
Intraoral myofascial sites (4-category variable)^†^	0.53	Moderate agreement
Jaw movements*	0.46	Acceptable agreement
*Joint pain*		
Palpation (4-category variable)^†^	0.67	Substantial agreement
Jaw movements*	0.96	Excellent agreement
*Range of motion*		
Vertical dimension (mm)^†^	0.68	Substantial agreement
Jaw excursions (mm)^†^	0.49	Moderate agreement
Jaw-opening pattern*	0.30	Regular agreement
*Joint sounds*		
Sound on jaw movement*	0.84	Excellent agreement

(Question) Symptom of TMD - Axis II*		
(3) Pain in facial area, the jaws or the jaw joint	0.81	Excellent agreement
(14a) Limitation in jaw opening	0.70	Good agreement
(14b) Diet restriction due to limitation in jaw opening	0.80	Good agreement

RDC/TMD, research diagnostic criteria for temporomandibular disorder

* Cohen's Kappa

† Intraclass correlation coefficient

### Criterion validity

Table 2 shows the correlations between the scores of the different subscales and variable pain, which was the sum of the positive responses to question number 3 of the RDC/TMD Axis II, *"Have you had pain in the face, jaw, temple, in front of the ear or in the ear in the past month?" *There were positive correlations between the CPQ_11-14 _total scores and variable pain (r = 0.32, p < 0.0001). Positive correlations were also observed between all of the domains of CPQ_11-14 _and pain scores. There were no significant correlations observed between the scale and subscale CPQ_8-10 _scores and variable pain, with the exception of the functional limitation subscale (r = 0.18, p < 0.05).

**Table 2:** Criterion validity: correlations between the CPQ scores and variable pain (Question 3, RDC/TMD Axis II) for TMD groups

TMD groups		Pain variable	
		
		**r**^**a**^	*P*
CPQ_8-10_	Total scale	0.14	0.089
*n = 141*	*Subscales*		
	Oral symptoms	0.13	0.106
	Functional limitations	0.18	0.024
	Emotional well-being	0.06	0.476
	Social well-being	0.09	0.278

CPQ_11-14_	Total scale	0.32	< 0.0001
*n = 176*	*Subscales*		
	Oral symptoms	0.33	< 0.0001
	Functional limitations	0.26	0.000
	Emotional well-being	0.24	0.001
	Social well-being	0.27	0.000

TMD, temporomandibular disorder; CPQ, child perceptions questionnaire

^a ^Spearman's correlation coefficient

### Discriminant construct validity

Children with signs and symptoms of TMD reported, on average, worse OHRQoL than the control group, as indicated by the mean overall scores of 20.6 versus 13.5, respectively (Table 3). The effect size of 0.43 indicated that the difference between the groups was moderate (p < 0.0001). The CPQ_8-10 _scores for the TMD group were also higher than in all subscales. When expressed as effect size, the magnitude of the mean differences was small to moderate. The mean score in the RDC/TMD diagnosis group (25.6 ± 22.3) was moderately higher than in the "supercontrol" group (7.5 ± 7.8) (Table 4). There were also significant differences between the groups for all the domains, with effect sizes ranging from moderate for functional (ES = 0.58), emotional (ES = 0.50) and social (ES = 0.54) domains to large for the oral symptom subscale (ES = 0.87).

**Table 3:** Discriminant construct validity: a comparison between the CPQ mean scores of the TMD and control groups

		*TMD group (n = 141)*	*Control group (n = 106)*		
				
		*Mean (SD)*	*Mean (SD)*	P*	***ES***^**†**^
CPQ_8-10_	Overall scale [0-100]	20.6 (17.7)	13.5 (15.4)	< 0.0001	0.43
	*Subscales*				
	Oral symptoms [0-20]	7.2 (4.0)	5.2 (3.9)	< 0.0001	0.55
	Functional limitations [0-20]	3.8 (4.2)	2.6 (3.8)	0.001	0.36
	Emotional well-being [0-20]	4.6 (4.7)	2.6 (4.1)	< 0.0001	0.52
	Social well-being [0-40]	5.5 (7.4)	3.1 (5.9)	0.009	0.39

		*TMD group (n = 176)*	*Control group (n = 124)*		
				
		*Mean (SD)*	*Mean (SD)*	P*	*ES*^†^

CPQ_11-14_	Overall scale [0-148]	27.6 (20.7)	16.3 (14.8)	< 0.0001	0.62
	*Subscales*				
	Oral symptoms [0-24]	7.0 (4.7)	5.2 (3.5)	< 0.0001	0.46
	Functional limitations [0-26]	6.5 (5.6)	3.6 (4.2)	< 0.0001	0.62
	Emotional well-being [0-36]	7.9 (7.6)	4.5 (5.6)	< 0.0001	0.53
	Social well-being [0-52]	5.9 (6.7)	2.9 (4.0)	< 0.0001	0.56

TMD, temporomandibular disorder; CPQ, child perceptions questionnaire

Values in square brackets indicate range of possible scores

* *P*-values obtained from Mann-Whitney test

† ES = Effect sizes, difference in group means/pooled SD

**Table 4:** Discriminant construct validity: CPQ overall and domain scores by the RDC/TMD diagnosis and "supercontrol" groups

		*RDC/TMD Diagnosis Group (n = 32)*	*Supercontrol Group (n = 28)*		
				
		*Mean (SD)*	*Mean (SD)*	P*	***ES***^**†**^
CPQ_8-10_	Overall scale [0-100]	25.6 (22.3)	7.5 (7.8)	< 0.0001	0.61
	*Subscales*				
	Oral symptoms [0-20]	8.7 (4.6)	3.5 (3.4)	< 0.0001	0.87
	Functional limitations [0-20]	4.8 (4.7)	1.3 (1.9)	< 0.0001	0.58
	Emotional well-being [0-20]	4.7 (5.2)	1.1 (1.7)	0.000	0.50
	Social well-being [0-40]	7.4 (9.6)	1.7 (3.1)	0.006	0.54

		*RDC/TMD Diagnosis Group (n = 69)*	*Supercontrol Group (n = 29)*		
				
		*Mean (SD)*	*Mean (SD)*	P*	*ES*^†^

CPQ_11-14_	Overall scale [0-148]	35.0 (24.1)	11.7 (9.6)	< 0.0001	0.88
	*Subscales*				
	Oral symptoms [0-24]	8.7 (5.8)	4.2 (2.1)	< 0.0001	0.74
	Functional limitations [0-26]	8.8 (7.0)	2.2 (2.9)	< 0.0001	0.89
	Emotional well-being [0-36]	10.0 (8.9)	3.1 (4.1)	< 0.0001	0.73
	Social well-being [0-52]	7.5 (6.8)	2.1 (3.4)	< 0.0001	0.82

TMD, temporomandibular disorder; CPQ, child perceptions questionnaire

Values in square brackets indicate range of possible scores

* *P*-values obtained from Mann-Whitney test

† ES = Effect sizes, difference in group means/pooled SD

Preadolescents in the TMD group had, on average, higher overall scores than in the control group (27.6 vs. 16.3; p < 0.0001) (Table 3). The same difference was observed in all domains, with the mean functional and social well-being score being two times higher in the former than in the latter patient group: 6.5 vs. 3.6 (p < 0.0001) and 5.9 vs. 2.9 (p < 0.0001). The magnitude of the differences between the clinical groups was moderate, ranging from 0.46 in the oral symptoms domain to 0.62 in the functional limitations domain. When the scores for the RDC/TMD diagnosis groups were examined, preadolescents diagnosed with TMD had significantly higher scores than the "supercontrol" group for all total and subscale CPQ_11-14 _scores (Mann-Whitney *U *test) (Table 4).

### Correlational construct validity

As an index of construct validity, Spearman's correlation was highly significant at the 0.0001 level in both global ratings for CPQ_8-10 _total scales in the TMD group (Table 5). Positive correlations were also observed between all the CPQ_8-10 _subscale scores and global oral health ratings, as well as overall well-being.

**Table 5:** Correlational construct validity: correlations between CPQ scores and global ratings of oral health and overall well-being (TMD groups)

TMD groups	CPQ_8-10 _(n = 141)				CPQ_11-14 _(n = 176)			
	
	Oral Health		Overall Well-being		Oral Health		Overall Well-being	
	
	**R**^**a**^	**P**^**b**^	**R**^**a**^	**P**^**b**^	**R**^**a**^	**P**^**b**^	**R**^**a**^	**P**^**b**^
Total scale	0.36	< 0.0001	0.41	< 0.0001	0.37	< 0.0001	0.62	< 0.0001
*Subscales*								
Oral symptoms	0.37	< 0.0001	0.39	< 0.0001	0.36	< 0.0001	0.42	< 0.0001
Functional limitations	0.25	0.002	0.41	< 0.0001	0.28	0.000	0.48	< 0.0001
Emotional well-being	0.44	< 0.0001	0.38	< 0.0001	0.34	< 0.0001	0.57	< 0.0001
Social well-being	0.28	0.000	0.36	< 0.0001	0.26	0.000	0.53	< 0.0001

TMD, temporomandibular disorder; CPQ, child perceptions questionnaire

The TMD group showed significant correlations between overall CPQ_11-14 _scores and global oral health ratings (p < 0.0001) and overall well-being (p < 0.0001). Significant correlations were also observed between the scores for all CPQ_11-14 _subscale scores and both global ratings (Table 5).

### Reliability

Internal consistency reliability was assessed for the TMD samples using Cronbach's alpha (Table 6). This was 0.93 for the total CPQ_8-10 _and ranged from 0.68 to 0.90 for the subscales, indicating an acceptable to good level of internal consistency. For the overall CPQ_8-10 _scale, the corrected item-total correlation coefficients were from 0.39 to 0.76 and for the domains the same coefficients ranged from 0.37 to 0.77. The alpha coefficients did not increase when any of the items were deleted.

**Table 6:** Internal consistency reliability: Cronbach's alpha, Alpha if item deleted and Corrected item-total correlation (TMD groups)

TMD groups		Number of items	Cronbach's alpha	Range of α's if items deleted	Range of corrected item total correlations
CPQ_8-10_	Total scale	25	0.93	(0.93-0.93)	(0.39-0.76)
*n = 141*	*Subscales*				
	Oral symptoms	5	0.68	(0.61-0.66)	(0.37-0.48)
	Functional limitations	5	0.78	(0.70-0.75)	(0.51-0.67)
	Emotional well-being	5	0.85	(0.81-0.83)	(0.60-0.71)
	Social well-being	10	0.90	(0.88-0.90)	(0.52-0.77)

CPQ_11-14_	Total scale	37	0.94	(0.93-0.94)	(0.28-0.73)
*n = 176*	*Subscales*				
	Oral symptoms	6	0.69	(0.62-0.68)	(0.33-0.51)
	Functional limitations	9	0.79	(0.76-0.78)	(0.40-0.57)
	Emotional well-being	9	0.90	(0.88-0.89)	(0.59-0.76)
	Social well-being	13	0.87	(0.85-0.87)	(0.28-0.67)

TMD, temporomandibular disorder; CPQ, child perceptions questionnaire

A total of 176 TMD individuals were used to test the internal reliability of the CPQ_11-14 _(Table 6). Cronbach's alpha for CPQ_11-14_, as a whole, was excellent (0.94). For the domains of the CPQ_11-14_, the coefficients ranged from 0.69 for oral symptoms to 0.90 for emotional well-being, indicating an acceptable to good levels of internal consistency reliability. The corrected item-total correlations for the total CPQ_11-14 _scale ranged from 0.28 to 0.73. For the CPQ_11-14 _subscales, the corrected item-total correlation coefficients ranged from 0.28, which represented the lower coefficient for the social well-being domain, to 0.76 for emotional well-being. The alpha was not higher when any item was deleted.

## Discussion

This study was undertaken to provide evidence of the reliability and validity of the CPQ_8-10 _and CPQ_11-14 _in children and preadolescents with signs and symptoms of TMD. Our previous study had indicated that these measures were able to discriminate between children and preadolescents with different levels of severity of dental caries, malocclusion, fluorosis and gingivitis [22]. According to Locker *et al. *[39], the process of evaluating HRQoL measures consists of two stages; the first involves an assessment of the reliability and validity and the second consists of on-going evaluations of the performance in different populations and the various contexts for which it was intended. Furthermore, the linguistic and cultural context in which a measure is used can have a bearing on the validity, as can the intended purpose of the measure; thus prior validity and reliability tests, the instruments must be translated, back-translated, and cross culturally adapted in order to ensure their conceptual and functional equivalences [22,27,31].

The RDC/TMD had been the best and most used classification system to date for epidemiological studies that sought to understand TMD etiology and mechanisms [40]. Together, Axis I and Axis II assessments constitute a comprehensive evaluation consistent with the biopsychosocial health model [2]. In this study, only three specific items for the latter were included, since they were more appropriate for the age sample. Accordingly, a questionnaire containing items regarding self-reported pain and associated symptoms of TMD [26] was used to replace the pain-related disability approach of RDC/TMD Axis II [41].

Reliability and validity are the basic underpinnings of any scientific measure. The reliability of a diagnostic instrument sets the upper limit for its validity [42]. Several studies evaluating the reliability of clinical findings have shown that the experiences and calibration of the examiners are crucial for accuracy of the results [32,43,44], as done in the present study. Individuals with most common TMD conditions as well as asymptomatic controls were included in the reliability assessment (n = 20) to ensure that a broad spectrum, ranging from none to severe findings, was present [32,45]. It provided a more realistically simulated actual clinical and research conditions, wherein patients and subjects who were both symptomatic and asymptomatic for TMD might actually appear to undergo RDC/TMD diagnostic examinations [46]. Other influencing factors included the feasibility of conducting such examinations in an acceptable time frame [46-48].

Considering the minimum acceptable level for agreement at 0.40 (kappa) for categorical measures and at 0.70 (ICC) for continuous variables [49], inconsistency was found in some RDC/TMD measurements, mainly in the pain scores and in the ranges of motion. However, the overall reliability results were still good. The poor intraoral muscle reliability found in the present study and by others [43,47] could be explained by the low specificity of muscle palpation [50,51]. Moreover, a low reproducibility for the pain scores is not unusual because pain intensities do vary over even short periods of time [52] partly due to poor memory recall for pain [53]. Only a moderate level of reproducibility was found for jaw excursions, compared with other studies where more agreement was observed [43,47]. In addition, differences in reliability findings may reflect variations in the methodology, such as differences in subject samples, numbers of examiners, study designs, statistical analyses, as well as prevalence and sampling variability [43,46,54].

Muscle tenderness was the most frequent clinical sign, found in 77.3% of children and 67% of preadolescents, agreeing with Tuerlings and Limme [55]. However, these results must be carefully considered given the low specificity of muscle palpation [50,51]. The prevalence of joint pain was substantial, being the second most frequent sign observed in 48.9% of the children and 44.9% of the preadolescents, higher than values observed in adolescents by Bonjardim *et al. *[41] (7.83%-10.6%). The less prevalent sign of TMD were TMJ sounds, found in just 5% of the children and 8% of the preadolescents and even lower than those observed in previous studies [41,56,57]. The difference in findings may reflect variations in the tools being used. The high sensitivity of RDC/TMD classification for TMJ sounds, which is based on reproducible clicks on two of three trials, contributes to the elimination of indistinct or temporary clicking sounds [32], decreasing the probability of false positive results.

In TMD groups, the presence of headaches was higher in children than in preadolescents, as previously observed [41,56,58]. There was no gender difference in the symptomatic children, but among preadolescents, the prevalence of headaches associated with TMD was higher in girls than in boys. In line with these findings, previous studies found an increasing of this association with age among adolescents, especially in females [59,60]. Similarly, the higher prevalence of the clinical signs of TMD, mainly painful signs among females, was consistent with some previous findings [57,58,61], whereas others found no gender-linked relationships [41,62]. The difference between genders could probably be explained by the fact that girls may be more sensitive to tenderness and pain on palpation of the TMJ and adjacent muscles [63] mainly in older age due to hormonal changes [56,61].

Ideally, criterion validity would be measured relative to a "gold standard." As no such standard exists for oral health status measures, criterion validity was evaluated by correlating the CPQ scores with a score corresponding to the sum of the answers to the item investigating pain (Question 3, RDC/TMD Axis II). This approach is consistent with literature reports that suggest the use of external criteria to test criterion validity [31]. Subjects with pain-associated conditions presented higher impacts on daily function in this study and in others performed in adult [3,12] and elderly [10] populations. Accordingly, the patients' well-being decreased as a function of pain duration and increased in pain intensity, frequency and number of pain sites [12,31]. In the only study to address this issue in youth patients, Jedel *et al. *[11] found that children and adolescents with TMD pain more than once a week were associated with higher impacts on physical functioning, emotional roles and behavioral roles, resulting in limitations on physical activities, school work and activities with friends. Similarly, positive correlations were observed between all the domains of CPQ_11-14 _and pain scores for preadolescents. Although a substantial prevalence of pain symptoms existed in the CPQ_8-10 _sample (36.2%), only the functional domain was associated with this variable. It is likely that reporting symptoms of minor severity or of fleeting nature resulted in such a high prevalence. Less severe pain and sensations may be responsible for less impaired OHRQoL in children reporting TMD. In fact, patients with TMD initially display functional limitations. These are followed by psychological discomfort, social disability and handicap and finally chronic pain [31]. This progression can also explain the different discriminant construct validity results, which compared the controls with both TMD groups and with the advanced cases.

The discriminant construct validity of the questionnaires was supported by their ability to detect differences in the impact on QoL, evidenced by the highest scores being seen in children and preadolescents with signs and symptoms of TMD. However, although the difference in scores supported the validity of the measures, the magnitude of these differences was only low to moderate. According to Reissmann *et al. *[14], the magnitude of TMD impact depends on the definition of the comparison group without TMD diagnoses. Although patients in the general population are the most plausible choice for comparison (which was chosen in the present study), they may have some signs and symptoms of TMD; these are insufficient to warrant an RDC/TMD diagnosis but sufficient to influence QoL. This is consistent with the findings by Reissmann *et al. *[14], where subjects without diagnosis had a more than 50% higher OHRQoL impact levels compared to subjects without any TMD sign or symptom. Other authors suggest that differences in scores of QoL measures can be properly interpreted only after minimally important differences have been recognized [64]. The minimum important difference is defined as the smallest difference in scores that patients perceive as being important, which would suggest a change in the patient's management [65]. This score can be determined only following longitudinal studies in which some individuals changed and some did not, either as the result of therapy or natural fluctuations in the disorder. This evaluation has yet to be undertaken with respect to the measures used in this study.

Evidence that the higher scores of the TMD individuals may be important was found in the responses of the advanced cases when compared to the "supercontrol" reports. Analyses of the scores derived from both questionnaires indicated that the QoL of children and preadolescents diagnosed with TMD was markedly worse than that of individuals with no current signs or symptoms of TMD. These results were consistent with the higher impact found in adults diagnosed with TMD when compared with control groups in the study by Rener-Sitar *et al. *[15], which suggested that diagnoses associated with pain (e.g., myofascial pain, arthralgia) have a higher impact than non-pain-related diagnoses (e.g., disc displacement with reduction). Considering that muscle tenderness was the most frequent diagnosis observed among the evaluated TMD sample, greater impact on QoL was expected for these subjects.

The construct validity was further supported when the CPQ scores were assessed for the TMD groups against the global questions, as high correlations between them suggest that they are measuring the same construct. Moreover, these associations showed that the reported issues and concerns of the TMD groups extend beyond oral health and are of sufficient magnitude to have some effect on their life as a whole. It means that the questionnaires actually measured as originally intended [38].

Accepted minimal standards for internal reliability coefficients are 0.70 for group comparisons and 0.90-0.95 for individual comparisons [66]. Accordingly, the reliability coefficients for both CPQ total and subscales exceeded standards for group and individual level comparisons [67], except for oral symptoms domains, which were slightly lower at 0.68 for CPQ_8-10 _and at 0.69 for CPQ_11-14_. However, these values can be acceptable, as they are far greater than 0.50, an indicative level for non-homogeneous scales [68]. According to Gherunpong *et al. *[59], alpha is not a perfect indicator of reliability, as it tends to underestimate the reliability of multidimensional scales and because lower values can be expected from health-related measures. All item-total correlations were above the minimum recommended level of 0.20 [19] and alpha did not increase when an item was deleted.

The greatest strenght of this study is the use of the standardized OHRQoL questionnaires and also the standardized assessment of the level of impairment of different anatomical structures that constitute a stomatognathic system according to the RDC/TMD protocol [15]. Besides that, the recruitment strategy of sample allowed for a spectrum of participants, which provided a valid estimation of the differences between individuals with variety levels of severity of the same clinical condition, so that a judgement could safely be made concerning the generalisation of the results to that population [31]. On the other hand, it is also important to recognize the limitations of the work performed in terms of the methodology and analytic strategies used [69]. Given the cross-sectional nature of the data study, the observed finding could address only the descriptive and discriminative potential of OHRQoL measures in relation to TMD condition. Further research is required to determine whether or not these instruments discriminated between groups of children and adolescents with different clinical conditions. Studies should also include the measurement of factors that may account for the variation in OHRQoL observed in TMD patients, as well as, for other oral conditions. Finally, longitudinal studies are required to demonstrate OHRQoL responsiveness to change prior to using it in a context where change is expected, desired or possible [70].

## Conclusions

The results of this study emphasize the importance of perceived health status and QoL assessment for evaluating TMD patients, since signs and symptoms of TMD can have a substantial functional, emotional and psychologic impact, negatively affecting the QoL of children and preadolescents. Comparisons between individuals with different levels of the same condition clearly indicated the progressive aspects of the pathology that appear in advanced cases. Sufficient descriptive and discriminative psychometric properties of CPQ in TMD populations make these instruments suitable for assessing OHRQoL in cross-sectional studies. Finally, further studies are required to confirm the evaluative potential of these measures in this clinical and age-specific population.

## Abreviattions

(CPQ): Child Perceptions Questionnaire; (CPQ_8-10_): Child Perceptions Questionnaire 8-10 years; (CPQ_11-14_): Child Perceptions Questionnaire 11-14 years; (*κ*): Cohen's Kappa; (ES): Effect size; (HRQoL): Health-related quality of life; (ICC): Intraclass correlation coefficient; (OHRQoL): Oral health-related quality of life; (QoL): Quality of life; (RDC/TMD): Research Diagnostic Criteria for temporomandibular disorder; (TMD): Temporomandibular disorder

## Competing interests

The authors declare that they have no competing interests.

## Authors' contributions

TSB participated in conception and design of the study, data analysis and interpretation, acquisition of data and drafting the manuscript. MSL contributed to the data collection. PMC made critical comments on the manuscript. MBDG participated in the conception and design of the study and critical revision of manuscript. All authors read and approved the final manuscript.

## Supplementary Material

Additional file 1 **Sample distribution in accordance with the evaluated characteristics - number of children (%)**. The data provided represent the distribution of the age-specific samples according to clinical groups, signs and symptoms of TMD and perception of oral health. Click here for file

## References

[B1] Suvinen TI Reade PC Kemppainen P Könönen M Dworkin SF Review of aetiological concepts of temporomandibular pain disorder: towards a biopsychosocial model for integration of physical disorder factors with psychological and psychosocial illness impact factors Eur J Pain 2005 9 613 633 10.1016/j.ejpain.2005.01.012 15978854

[B2] Gatchel RJ Peng YB Peters ML Fuchs PN Turk DC The biopsychosocial approach to chronic pain: scientific advances and future directions Psychol Bull 2007 133 581 624 17592957 10.1037/0033-2909.133.4.581

[B3] Barros de VM Seraidarian PI Côrtes MI de Paula LV The impact of orofacial pain on the quality of life of patients with temporomandibular disorder J Orofac Pain 2009 23 28 37 19264033

[B4] Barbosa S Tde Miyakoda LS Pocztaruk L Rde Rocha CP Gavião MB Temporomandibular disorders and bruxism in childhood and adolescence: review of the literature Int J Pediatr Otorhinolaryngol 2008 72 299 314 10.1016/j.ijporl.2007.11.006 18180045

[B5] Castelo PM Gavião MB Pereira LJ Bonjardim LR Relationship between oral parafunctional/nutritive sucking habits and temporomandibular joint dysfunction in primary dentition Int J Paediatr Dent 2005 15 29 36 10.1111/j.1365-263X.2005.00608.x 15663442

[B6] Pereira LJ Pereira-Cenci T Del Bel Cury AA Pereira SM Pereira AC Ambosano GM Gavião MB Risk indicators of temporomandibular disorder incidences in early adolescence Pediatr Dent 2010 32 324 328 20836952

[B7] Gonçalves DA Dal Fabbro AL Campos JA Bigal ME Speciali JG Symptoms of temporomandibular disorders in the population: an epidemiological study J Orofac Pain 2010 24 270 278 20664828

[B8] Farsi N Alamoudi N Feteih R El-Kateb M Association between temporo mandibular disorders and oral parafunctions in Saudi children Odontostomatol Trop 2004 27 9 14 15536715

[B9] Dahlström L Carlsson GE Temporomandibular disorders and oral health-related quality of life. A systematic review Acta Odontol Scand 2010 68 80 85 10.3109/00016350903431118 20141363

[B10] Graue M Wentzel-Larsen T Hanestad BR Båtsvik B Søvik O Measuring self-reported, health-related, quality of life in adolescents with type 1 diabetes using both generic and disease-specific instruments Acta Paediatr 2003 92 1190 1196 10.1111/j.1651-2227.2003.tb02483.x 14632337

[B11] Jedel E Carlsson J Stener-Victorin E Health-related quality of life in child patients with temporomandibular disorder pain Eur J Pain 2007 11 557 563 10.1016/j.ejpain.2006.07.007 16935534

[B12] Murray H Locker D Mock D Tenenbaum HC Pain and the quality of life in patients referred to a craniofacial pain unit J Orofac Pain 1996 10 316 323 9161236

[B13] Locker D Grushka M The impact of dental and facial pain J Dent Res 1987 66 1414 1417 10.1177/00220345870660090101 3476612

[B14] Reissmann DR John MT Schierz O Wassell RW Functional and psychosocial impact related to specific temporomandibular disorder diagnoses J Dent 2007 35 643 650 10.1016/j.jdent.2007.04.010 17573174

[B15] Rener-Sitar K Celebić A Stipetić J Marion L Petricević N Zaletel-Kragelj L Oral health related quality of life in Slovenian patients with craniomandibular disorder Coll Antropol 2008 32 513 517 18756903

[B16] Luo Y McMillan AS Wong MC Zheng J Lam CL Orofacial pain conditions and impact on quality of life in community-dwelling elderly people in Hong Kong J Orofac Pain 2007 21 63 71 17312643

[B17] Jokovic A Locker D Stephens M Kenny D Tompson B Guyatt G Validity and reliability of a questionnaire for measuring child oral-health-related quality of life J Dent Res 2002 81 459 463 10.1177/154405910208100705 12161456

[B18] Jokovic A Locker D Tompson B Guyatt G Questionnaire for measuring oral health-related quality of life in eight- to ten year- old children Pediatr Dent 2004 26 512 518 15646914

[B19] Gherunpong S Tsakos G Sheiham A Developing and evaluating an oral health-related quality of life index for children; The CHILD-OIDP Community Dental Health 2004 21 161 169 15228206

[B20] Pahel BT Rozier RG Slade GD Parental perceptions of children's oral health: the Early Childhood Oral Health Impact Scale (ECOHIS) Health Qual Life Outcomes 2007 5 6 10.1186/1477-7525-5-6 17263880 PMC1802739

[B21] Broder HL Wilson-Genderson M Reliability and convergent and discriminant validity of the Child Oral Health Impact Profile (COHIP Child's version) Community Dent Oral Epidemiol 2007 35 20 31 10.1111/j.1600-0528.2007.0002.x 17615047

[B22] Barbosa TS Tureli MCM Gavião MBD Validity and reliability of the Child Perceptions Questionnaires applied in Brazilian children BMC Oral Health 2009 9 13 10.1186/1472-6831-9-13 19450254 PMC2696414

[B23] Sheiham A Maizels JE Cushing AM The concept of need in dental care Int Dent J 1982 32 265 270 6958655

[B24] Locker D Health outcomes of oral disorders Int J Epidemiol 1995 24 85 89 10.1093/ije/24.supplement_1.s85 7558559

[B25] Pereira LJ Pereira-Cenci T Pereira SM Cury AA Ambrosano GM Pereira AC Gavião MB Psychological factors and the incidence of temporomandibular disorder in early adolescence Braz Oral Res 2009 23 155 160 10.1590/S1806-83242009000200011 19684950

[B26] Riolo ML Brandt D TenHave TR Associations between occlusal characteristics and signs and symptoms of TMJ dysfunction in children and young adults Am J Orthod Dentofacial Orthop 1987 92 467 477 10.1016/0889-5406(87)90228-9 3500634

[B27] de Lucena LB Kosminsky M da Costa LJ de Góes PS Validation of the Portuguese version of the RDC/TMD Axis II questionnaire Braz Oral Res 2006 20 312 317 10.1590/S1806-83242006000400006 17242791

[B28] Dworkin SF LeResche L Research diagnostic criteria for temporomandibular disorder: review, criteria, examinations and specifications, critique J Craniomandib Disord 1992 6 301 355 1298767

[B29] Pereira FJ Favilla EE Dworkin SF Huggins KH Critérios de diagnóstico para pesquisa das desordens temporomandibulares RDC/TMD http://www.rdc-tmdinternational.org/OtherResources/TranslationGuidelines.aspx

[B30] Bonjardim LR Gaviao MB Carmagnani FG Pereira LJ Castelo PM Signs and symptoms of temporomandibular joint dysfunction in children with primary dentition J Clin Pediatr Dent 2003 28 53 58 14604143 10.17796/jcpd.28.1.0772w75g91963670

[B31] Segù M Collesano V Lobbia S Rezzani C Cross-cultural validation of a short form of the Oral Health Impact Profile for temporomandibular disorder Community Dent Oral Epidemiol 2005 33 125 130 10.1111/j.1600-0528.2005.00215.x 15725175

[B32] Dworkin SF LeResche L DeRouen T Von Korff M Assessing clinical signs of temporomandibular disorder: reliability of clinical examiners J Prosthet Dent 1990 63 574 579 10.1016/0022-3913(90)90079-R 2338670

[B33] Shrout PE Fleiss JL Intraclass correlation: uses in assessing rater reliability Psych Bull 1979 86 420 428 10.1037//0033-2909.86.2.420 18839484

[B34] Kramer MS Feinstein AR The biostatistics of concordance Clin Pharmacol Ther 1981 29 111 123 10.1038/clpt.1981.18 7460469

[B35] Cohen J A coefficient of agreement for nominal scales Educ Psychol Measurement 1960 20 37 46 10.1177/001316446002000104

[B36] Cohen J Statistical Power Analysis for the Behavioural Sciences 1988 Hillside, NJ: Lawrence Erlbaum Associates

[B37] Cronbach LJ Coefficient alpha and the internal structure of tests Psychometrika 1951 16 297 334 10.1007/BF02310555

[B38] Streiner DL Norman GR A practical guide to their development and use 2000 New York: Oxford University Press Health measurement scales

[B39] Locker D Jokovic A Tompson B Prakash P Is the Child Perceptions Questionnaire for 11-14 year olds sensitive to clinical and self-perceived variations in orthodontic status? Community Dent Oral Epidemiol 2007 35 179 185 10.1111/j.1600-0528.2006.00324.x 17518964

[B40] International Consortium for RDC/TMD-based Research http://www.rdc-tmdinternational.org

[B41] Bonjardim LR Gavião MB Pereira LJ Castelo PM Garcia RC Signs and symptoms of temporomandibular disorder in adolescents Braz Oral Res 2005 19 93 98 10.1590/S1806-83242005000200004 16292440

[B42] Smith TW Friedman HS, Silver RC Measurement in health psychology research Foundations of Health Psychology 2007 New York: Oxford University 19 51

[B43] List T John MT Dworkin SF Svensson P Recalibration improves inter-examiner reliability of TMD examination Acta Odontol Scand 2006 64 146 152 10.1080/00016350500483137 16809191

[B44] Steenks MH de Wijer A Validity of the Research Diagnostic Criteria for Temporomandibular Disorder Axis I in clinical and research settings J Orofac Pain 2009 23 9 16 19264032

[B45] Lobbezoo F van Selms MK John MT Huggins K Ohrbach R Visscher CM van der Zaag J van der Meulen MJ Naeije M Dworkin SF Use of the Research Diagnostic Criteria for Temporomandibular Disorder for multinational research: translation efforts and reliability assessments in The Netherlands J Orofac Pain 2005 19 301 308 16279481

[B46] John MT Dworkin SF Mancl LA Reliability of clinical temporomandibular disorder diagnoses Pain 2005 118 61 9 10.1016/j.pain.2005.07.018 16154702

[B47] Wahlund K List T Dworkin SF Temporomandibular disorder in children and adolescents: reliability of a questionnaire, clinical examination, and diagnosis J Orofac Pain 1998 12 42 51 9656898

[B48] White P Lewith G Prescott P The core outcomes for neck pain: validation of a new outcome measure Spine 2004 29 1923 1930 10.1097/01.brs.0000137066.50291.da 15534418

[B49] Look JO John MT Tai F Huggins KH Lenton PA Truelove EL Ohrbach R Anderson GC Shiffman EL The Research Diagnostic Criteria For Temporomandibular Disorder. II: reliability of Axis I diagnoses and selected clinical measures J Orofac Pain 2010 24 25 34 20213029 PMC3098131

[B50] Stratmann U Mokrys K Meyer U Kleinheinz J Joos U Dirksen D Bollmann F Clinical anatomy and palpability of the inferior lateral pterygoid muscle J Prosthet Dent 2000 83 548 554 10.1016/S0022-3913(00)70013-8 10793387

[B51] Türp JC Minagi S Palpation of the lateral pterygoid region in TMD - where is the evidence? J Dent 2001 29 475 483 10.1016/S0300-5712(01)00042-2 11809325

[B52] Ohrbach R Turner JA Sherman JJ Mancl LA Truelove EL Schiffman EL Dworkin SF The Research Diagnostic Criteria for Temporomandibular Disorder. IV: evaluation of psychometric properties of the Axis II measures J Orofac Pain 2010 24 48 62 20213031 PMC3115780

[B53] Jamison RN Sbrocco T Parris WC The influence of physical and psychosocial factors on accuracy of memory for pain in chronic pain patients Pain 1989 37 289 294 10.1016/0304-3959(89)90193-0 2755710

[B54] Sousa LM Nagamine HM Chaves TC Grossi DB Regalo SC Oliveira AS Evaluation of mandibular range of motion in Brazilian children and its correlation to age, height, weight, and gender Braz Oral Res 2008 22 61 66 10.1590/S1806-83242008000100011 18425247

[B55] Tuerlings V Limme M The prevalence of temporomandibular joint dysfunction in the mixed dentition Eur J Orthod 2004 26 311 320 10.1093/ejo/26.3.311 15222717

[B56] Farsi NM Symptoms and signs of temporomandibular disorder and oral parafunctions among Saudi children J Oral Rehabil 2003 30 1200 1208 10.1111/j.1365-2842.2003.01187.x 14641664

[B57] Sönmez H Sari S Oksak G Oray Camdeviren H Prevalence of temporomandibular dysfunction in Turkish children with mixed and permanent dentition J Oral Rehabil 2001 28 280 285 10.1111/j.1365-2842.2001.tb01678.x 11394375

[B58] Thilander B Rubio G Pena L de Mayorga C Prevalence of temporomandibular dysfunction and its association with malocclusion in children and adolescents: an epidemiologic study related to specified stages of dental development Angle Orthod 2002 72 146 154 11999938 10.1043/0003-3219(2002)072<0146:POTDAI>2.0.CO;2

[B59] LeResche L Mancl LA Drangsholt MT Saunders K Korff MV Relationship of pain and symptoms to pubertal development in adolescents Pain 2005 118 201 209 10.1016/j.pain.2005.08.011 16213087

[B60] Nilsson IM List T Drangsholt M Prevalence of temporomandibular pain and subsequent dental treatment in Swedish adolescents J Orofac Pain 2005 19 144 150 15895837

[B61] List T Wahlund K Wenneberg B Dworkin SF TMD in children and adolescents: prevalence of pain, gender differences, and perceived treatment need J Orofac Pain 1999 13 9 20 10425964

[B62] Nilsson IM Drangsholt M List T Impact of temporomandibular disorder pain in adolescents: differences by age and gender J Orofac Pain 2009 23 115 122 19492536

[B63] Dao TT LeResche L Gender differences in pain J Orofac Pain 2000 14 169 184 11203754

[B64] Juniper G Guyatt G Willan A Griffith L Determining a minimal important change in a disease-specific quality of life questionnaire J Clin Epidemiol 1994 47 81 87 10.1016/0895-4356(94)90036-1 8283197

[B65] Jaeschke R Singer J Guyatt G Measurement of health status: ascertaining the minimal clinically important difference Controlled Clin Trials 1989 10 407 415 10.1016/0197-2456(89)90005-6 2691207

[B66] Scientific Advisory Committee of the Medical Outcomes Trust Assessing health status and quality-of-life instruments: attributes and review criteria Qual Life Res 2002 11 193 205 10.1023/A:1015291021312 12074258

[B67] Norman GR Streiner DL PDQ statistics 1996 Louis: Mosby

[B68] Bowling A Research Methods in Health: Investigating Health and Health Services 1997 Buckingham: Open University Press

[B69] Li S Veronneau J Allison PJ Validation of a French language version of the Early Childhood Oral Health Impact Scale (ECOHIS) Health Qual Life Outcomes 2008 6 9 10.1186/1477-7525-6-9 18211711 PMC2245912

[B70] Li S Malkinson S Veronneau J Allison PJ Testing responsiveness to change for the early childhood oral health impact scale (ECOHIS) Community Dent Oral Epidemiol 2008 36 542 548 10.1111/j.1600-0528.2008.00434.x 18422706

