# Supplementary material for: Evaluating oral health-related quality of life measure for children and preadolescents with temporomandibular disorder
Source: Health Qual Life Outcomes. 2011 May 12;9:32. doi: 10.1186/1477-7525-9-32 (PMC3115836; doi:10.1186/1477-7525-9-32)
Supplement: Additional file 1 — Sample distribution in accordance with the evaluated characteristics - number of children (%). The data provided represent the distribution of the age-specific samples according to clinical groups, signs and symptoms of TMD and perception of oral health. [file 1477-7525-9-32-S1.PDF]

Table 1. Sample distribution in accordance with the evaluated characteristics - number of children (%)

	Children			*Preadolescents*		
	Boys	Girls	Total	Boys	Girls	Total
Number (%)[†]	113 (45.7)	134 (54.3)	247 (100.0)	122 (40.7)**	178 (59.3)**	300 (100.0)
Mean age + SD	9.0±0.8	9.1 ±0.8	9.0±0.8	12.2±1.2	12.1±1.1	12.1±1.1
Clinical groups[†]						
Group control	58 (54.7)	48 (45.3)	106 (42.9)	60 (48.4)	64 (51.6)	124 (41.3)
Group TMD	55 (39.0)*	86 (61.0)*	141 (57.1)	62 (35.2)**	114 (64.8)**	176 (58.7)
RDC/TMD diagnostic groups[†§]						
Group I: muscle disorder	5 (19.2)**	21 (80.8)**	26 (18.4)	12 (27.9)*	31 (72.1)*	43 (24.4)
Group II: disc displacements	0 (0.0)	1 (100.0)	1 (0.7)	0 (0.0)	5 (100.0)	5 (2.8)
Group III: arthralgia, arthritis, arthrosis	2 (40.0)	3 (60.0)	5 (3.5)	6 (28.6)	15 (71.4)	21 (11.9)
Symptoms of TMD (self-report questionnaire)[†§]						
Facial/jaw pain	14 (27.5)*	37 (72.5)*	51 (36.2)	23 (31.5)*	50 (68.5)*	73 (41.5)
Difficult in opening	16 (37.2)	27 (62.8)	43 (30.5)	16 (28.1)*	41 (71.9)*	57 (32.4)
Joint sounds	21 (46.7)	24 (53.3)	45 (31.9)	16 (41.0)	23 (59.0)	39 (22.2)
Teeth grinding	13 (29.5)*	31 (70.5)*	44 (31.2)	15 (34.9)	28 (65.1)	43(24.4)
Headache	34 (37.4)	57 (62.6)	91 (64.5)	42 (32.6)**	87 (67.4)**	129 (73.3)
Signs of TMD (RDC/TMD Axis I)[†§]						
Muscle tenderness	41 (37.6)*	68 (62.4)*	109 (77.3)	39 (33.1)**	79 (66.9)**	118 (67.0)

Joint pain	22 (31.9)*	47 (68.1)*	69 (48.9)	24 (30.4)**	55 (69.6)**	79 (44.9)
Limited mouth opening	12 (28.6)*	30 (71.4)*	42 (29.8)	17 (40.5)	25 (59.5)	42 (23.9)
Deviation in jaw excursions	18 (50.0)	18 (50.0)	36 (25.5)	16 (30.2)*	37 (69.8)*	53 (30.1)
TMJ sounds	2 (40.0)	3 (60.0)	5 (3.5)	2 (14.3)	12 (85.7)	14 (8.0)
Perception of oral health						
Mean CPQ score ± SD‡	14.7±16.7***	19.1±17.1***	17.5±17.1	20.4±18.0	22.6±20.0	22.9±19.3

TMD, temporomandibular disorder; TMJ, temporomandibular joint; CPQ, child perceptions questionnaire

§ Results of TMD samples

† Chi-square test; ‡ Mann-Whitney test

*p<0.05; **p<0.01; *** p<0.001
